# Determinants of factors affecting readiness of academic institutions to conduct knowledge translation in low- and middle-income countries

**DOI:** 10.3389/fpubh.2023.1302756

**Published:** 2024-01-08

**Authors:** Anna Kalbarczyk, Aditi Rao, Olakunle O. Alonge

**Affiliations:** Department of International Health, Johns Hopkins Bloomberg School of Public Health, Baltimore, MD, United States

**Keywords:** knowledge translation, organizational readiness, barriers, determinants, capacity building

## Abstract

**Introduction:**

Capacity building strategies have been used to improve uptake of knowledge translation (KT) activities among academic institutions, but little is known about their effectiveness, contextual responsiveness, and adaptability. Many of these strategies target individuals while few address institutional gaps. This research describes the determinants for conducting KT (or readiness to conduct such activities) at the institutional level across diverse LMIC contexts to inform the development of capacity building strategies.

**Methods:**

We conducted a survey to assess organizational readiness to conduct KT to public health researchers and practitioners from six academic institutions in Bangladesh, Ethiopia, DRC, India, Indonesia and Nigeria and members of a global knowledge-to-action working group. We assessed the frequency of barriers and facilitators to KT and their relationship to age, gender, country, and KT experience. We then performed logistic regression to identify determinants of five underlying factors demonstrated to influence KT readiness in LMICs (Institutional Climate, Organization Change Efficacy, Prioritization and Cosmopolitanism, Self-Efficacy and Financial Resource) along with their composite score, which represented an overall readiness score to conduct KT.

**Results:**

A total of 111 responses were included in the final analysis. Participants represented 10 LMICs; a majority were 30–49 years old (57%) and most were male (53%). Most participants had professional foci in research (84%), teaching (62%), and project coordination (36%) and 59% indicated they had experience with KT. Common facilitators included motivated faculty (57%) and dedicated personnel (40%). Funding (60%), training (37%), and time (37%) were the most frequently reported barriers. In the adjusted model, age, gender, country, and professional focus were significantly associated with at least one factor. Prior experience with KT was significantly and positively (OR = 9.07; CI: 1.60–51.58; *p* < 0.05) associated with the overall KT readiness to conduct KT.

**Discussion:**

Different KT readiness factors are relevant for younger (institutional climate) vs. older (self-efficacy) academic professionals, suggesting value in cross-generational collaborations. Leadership and gender were both relevant for organizational change efficacy indicating a need to engage leaders and promote women to influence organizational change. Institutions in different countries may be at different stages of change; readiness assessments can be used to systematically identify needs and develop targeted strategies.

## 1 Introduction

Bridging the gap between what we know through research and what we do in policy and practice, aka the “know-do gap” has been called “one of the most important challenges for public health in this century” (1). Knowledge generated through rigorous and ethically sound research processes can improve health outcomes and strengthen health systems (2, 3) but evidence shows limited translation and uptake of research knowledge by policy makers in different settings (4). The translation, uptake, and utilization of this knowledge is imperative for low-and middle-income countries (LMICs) which face the highest burdens of disease and have the fewest resources.

Knowledge Translation (KT) is a broad term used to describe the process of generating and using evidence to improve programs and policies and create action, and academic institutions have a major role to play in KT activities across different settings (5). Several studies have documented the unique barriers academic institutions face in conducting KT activities in high-income countries (5–10). While these barriers have been less well-documented in LMICs, some research has shown a lack of knowledge of KT and how to do it, limited institutional support (e.g., infrastructure, staffing, incentives), competing priorities, and cost of KT activities, are important barriers to KT in low resource settings (11). Limitations to conduct KT in LMICs may also be related to global power imbalances (i.e., perceptions that knowledge generated from LMICs is less relevant and credible) and misalignment between research and national priorities (12).

Organizational readiness is a multi-level, multi-faceted construct influenced by internal and external contexts, that highlights that implementing organizational change requires collective action (13). Organizational readiness assessments to conduct KT (such as the OR4KT) are one approach to diagnose and further explore these barriers; they can also be used to prioritize areas for growth, change, and capacity building (14–16). However, the factors that affect readiness for change are context dependent (13, 17); that is, the factors that influence readiness of academic institutions to participate in knowledge generation, synthesis, use, and uptake in one setting may not be the same in another. Thus, it is important to unpack the influence of various contextual factors on the readiness to do KT, and such understanding will further contribute to efforts and strategies for addressing barriers to KT among academic institutions in LMICs.

There is a disconnect between current readiness tools used for KT and linkages to the identification and implementation of strategies to improve KT. Further, there is limited empirical evidence on the effectiveness of strategies to improve KT and how they can be translated to different settings (18, 19). While we know training and technical assistance can build knowledge and skills, it is less clear how these and other approaches affect or are affected by the organization (19). Capacity building efforts also tend to focus on the individual, rather than the larger institution or contextual environment (20). While this approach continues to face significant criticism (21, 22), well-documented, systems-level capacity building interventions, particularly for institutions in LMICs, remain less established largely because of a limited understanding of how these interventions address specific barriers and determinants of those barriers at the institutional level. A recent literature review on KT for public health in LMICs found consistent support for including institutional strengthening activities for KT (23) but noted that existing approaches are sometimes *ad hoc* (24) and remain targeted at individuals (25, 26).

To effectively implement capacity building strategies to improve readiness and KT interventions at any level, it is important to understand underlying determinants that influence the effectiveness of such interventions (27). While KT readiness itself may not be readily changed, the variables that influence readiness may be more clearly targeted and influenced. By identifying determinants of KT readiness we can inform the development of well-defined, reproduceable strategies to build capacity of academic institutions to conduct KT in low-resource settings.

This research is embedded in a 5-year parent project, “Synthesis and Translation of Research and Innovations from Polio Eradication” (STRIPE), designed to map, synthesize, and disseminate knowledge from polio eradication (28). The academic institutional members of the consortium, based in six LMICs including Bangladesh, Democratic Republic of the Congo (DRC), Ethiopia, India, Indonesia, and Nigeria, were the focus of this study.

## 2 Methods

This research seeks to quantify the determinants for conducting KT activities (or readiness to conduct such activities) across diverse LMIC contexts, especially mapped to the institutional level. We use the Canadian Institutes of Health Research's definition of KT – “A dynamic and iterative process that includes the synthesis, dissemination, exchange, and ethically sound application of knowledge to improve health, provide more effective health services, and strengthen the health care system” (29). This definition informed our conception of KT and analytical interpretation. We applied a validated tool for assessing readiness to conduct KT among academic institutions in LMICs, estimated readiness score and identified determinants of the readiness scores at the individual and institutional levels (30). It is hoped that such assessment will lead to the development of comprehensive capacity building strategies tailored to these determinants and responsive to LMIC contexts.

The research reported in this paper is part of an exploratory-sequential mixed methods study designed to develop capacity building strategies to support academic institutions to conduct KT. During the first Phase, we conducted a literature review and a series of key-informant interviews (KIIs) with institutional and government leaders in each of the six STRIPE countries to identify key barriers, facilitators, and strategies to address individual and institutional readiness to conduct KT.

### 2.1 Study population and procedure

The study population included faculty, staff, and leadership at each of the six academic STRIPE institutions in addition to members of the Translating Evidence to Action Working Group (TWG) within the Health Systems Global consortium. The TWG is comprised of ~220 members, representing researchers, implementers, and policymakers with a focus on translation of evidence into action. We know anecdotally that members are largely based in LMICs and have substantial expertise in KT. We also know that given the complexities of KT, faculty do not work alone, and require support from leaders and a variety of staff with different disciplinary foci (11). Therefore, we felt it was important to capture the experiences of all members of the academic community who may be regularly involved in KT.

STRIPE primary investigators at each institution worked with the research team to identify and distribute the survey to members of their institution who had conducted KT activities, been members of KT teams, and/or served as leaders in the institution. To be eligible for the survey individuals must be active members of an academic institution within the STRIPE consortium or the TWG and the institution must be based in an LMIC. A total of 200 eligible participants were identified across the STRIPE consortium and invited to participate in the survey. The TWG does not maintain demographic information on its members; therefore, it is unclear how many of the 220 members were eligible to complete the survey.

The survey was distributed online via Qualtrics, an online survey software, with options to respond in English or French. Surveys were circulated via email through STRIPE PI's, members of their institutions' leadership, or through local research assistants. The TWG membership list serve was used to contact members and recruit participants. Survey data was collected from February 06, 2020–March 25, 2020.

### 2.2 Analysis

We conducted Exploratory Factor Analysis (EFA) to identify five factors of readiness for individuals and institutions in LMICs to conduct KT as previously reported (1) Institutional Climate, (2) Organization Change Efficacy, (3) Prioritization and Cosmopolitanism, (4) Self-efficacy, and (5) Financial Resources (29). Based on the factor analysis, a KT readiness index score was estimated for each individual included in the study population. To do this, factor loadings for items grouped under a specific factor were used to a predict factor score and these were aggregated across the five factors for each individual to estimate the KT readiness score (30). While some items reflect institutional characteristics, all were aggregated at the individual level.

### 2.3 Dependent variable – KT index

The predicted factor scores were created using the least squares regression approach to predict each individual's placement on the construct based on the value of their response to the relevant items and the items' factor loadings for that construct. The least squares regression approach to predicting factor scores is a refined computation method, which produces factor scores that are linear combinations of the observed variables, considering shared variance as well as error term variance (30). This approach was chosen, over other non-refined methods, as it is more exact and provides estimates that are standardized scores, as well as maximizing validity by retaining relationships between factors, i.e., correlations among factors scores remain the same as the correlations among factors, thus obtaining unbiased estimates of the true factor scores (31).

The factor scores and KT index were then transformed into binary variables, using their median values as the cut-off. We explored other operations (e.g., treating as continuous variables, using deciles and dichotomizing based on the mean), and settled on median cut-off given that it retained the information in the data (similar to treating as continuous variables or deciles) while simplifying the interpretation of our outcome variables (e.g., each of the five constructs and KT index). Logistic regression analyses were conducted to identify the determinants for each construct and KT index.

### 2.4 Explanatory variables

Explanatory variables assessed in these regression models include *age* (18–29, 30–49, 50–69, 70+ years), *gender* (male, female), *country* (Bangladesh, DRC, Ethiopia, India, Indonesia, Nigeria, Others), *experience with KT* (no, yes, unsure), and *professional focus* (research, administrative, leadership, coordination, communications, external affairs, development, management, teaching, finance, it, regulatory services, other). For the KT index regression model, we also included variables on the *facilitators for conducting KT* (dedicated personnel, funding, protected time, training, institutional mission, relations with the ministry, institutional rewards, motivated faculty, other) and *barriers to conducting KT* (funding, training, time, networks with the ministry, awareness, interest from policymakers, understanding policy, experience with KT, incentives, leadership, other). These facilitators/barriers variables were assessed at the institutional level by asking individual respondents to reflect on their institutions and respondents could select more than one listed facilitator/barrier.

Bivariate analysis was conducted to estimate the association between each of the factors and explanatory variables, as well as multivariate analysis to estimate the independent effect of each explanatory variable, adjusting for all others. Final models for each factor were determined based on checks for collinearity [using the variance inflation factor (vif) metric, where we ensured a vif value of 1–5], the likelihood ratio test, and the Hosmer–Lemeshow test for goodness of fit. All variables were not included in the final models.

### 2.5 Regression analysis

Regression models were developed separately for KT index as an outcome and each of the five factors as outcomes. In developing regression models for each factor, we also explored interactions between variables to ascertain any confounding or modifying effects. However, we did not include any interactions in the final model due to the small sample size of participants.

Univariate associations between demographic characteristics (age, gender, country, and past experience with KT) and the reported barriers and facilitators to KT activities were assessed using the Kruskal–Wallis test. A *p* ≤ 0.05 was regarded as statistically significant. All statistical analysis was performed using STATA (version 13).

### 2.6 Summary of the tool

We developed a survey tool based on a review of validated readiness tools identified in the literature review and findings from the KIIs which are described extensively in Kalbarczyk et al. (11, 30). We then completed exploratory factor analysis to identify five factors as essential for assessing readiness to do KT in LMICs: (F1) Institutional Climate (how individuals perceive and describe their work setting), (F2) Organization Change Efficacy (organizations' members shared beliefs in their collective capabilities), (F3) Prioritization and Cosmopolitanism (internal and external institutional networks and priorities), (F4) Self-efficacy (an individual's belief in their own abilities), and (F5) Financial Resources (availability of internal and external monies to conduct KT), and then assessed the psychometric properties of items for measuring these factors (30). The internal consistencies of items for these factors ranged from 0.52 to 0.78, and the details and additional results on the psychometric assessments are described in Kalbarczyk et al. (30). The final tool included items for measuring each of the five factors and a set of eight demographic questions – 76 statements/questions altogether and participants were asked to assess the extent to which they agreed with statements using a five-point Likert scale (“strongly agree” to “strongly disagree”). Participants were also asked to indicate the top 3 barriers and facilitators to conducting KT in their setting; barriers and facilitators were provided as a list based on findings from the literature and the interviews.

This research was declared “Non-Human Subjects” (because we asked individuals about their professional, not personal experiences) by the Johns Hopkins Bloomberg School of Public Health Institutional Review Board on October 08, 2019.

## 3 Results

We received 158 responses to the survey across the 6 STRIPE institutions and TWG. Forty-seven respondents completed 9% or less of the survey (e.g., only consented or provided some demographic information) and these responses were dropped. Dropped responses were reviewed to ensure they did not cluster by country, or any other demographic information provided. A total of 111 responses were included in the final analysis.

Participant characteristics are displayed in Table 1. Participants from the STRIPE institutions were based in Bangladesh (*n* = 27), Indonesia (*n* = 19), Nigeria (*n* = 16), India (*n* = 16), DRC (*n* = 12), and Ethiopia (*n* = 11). Ten individuals from TWG participated in the survey from 5 other countries including Colombia, Georgia, Rwanda, South Africa, and Uganda. A majority of respondents (*n* = 64, 57.66%) were 30–49 years old. Most participants were male (*n* = 59, 53.15%).

**Table 1:** Respondent characteristics.

**Variable**	**Bangladesh**	**DRC**	**Ethiopia**	**India**	**Indonesia**	**Nigeria**	**Others**	**Total**
	***n** =* **27 (%)**	***n** =* **12 (%)**	***n** =* **11 (%)**	***n** =* **16 (%)**	***n** =* **19 (%)**	***n** =* **16 (%)**	***n** =* **10 (%)**	***n** =* **111 (%)**
**Age**								
18–29 years	13 (48.15)	0 (0.00)	0 (0.00)	0 (0.00)	6 (31.58)	0 (0.00)	0 (0.00)	19 (17.12)
30–49 years	12 (44.44)	6 (50.00)	8 (72.73)	7 (43.75)	11 (57.89)	12 (75.00)	8 (80.00)	64 (57.66)
50–69 years	2 (7.41)	5 (41.67)	3 (27.27)	7 (43.75)	0 (0.00)	3 (18.75)	2 (20.00)	22 (19.82)
≥70 years	0 (0.00)	1 (8.33)	0 (0.00)	0 (0.00)	0 (0.00)	0 (0.00)	0 (0.00)	1 (0.90)
**Gender**								
Male	16 (59.26)	6 (50.00)	8 (72.73)	10 (62.50)	5 (26.32)	9 (56.25)	5 (50.00)	59 (53.15)
Female	9 (33.33)	5 (41.67)	3 (27.27)	6 (37.50)	14 (73.68)	7 (43.75)	4 (40.00)	48 (43.24)
**Professional focus**								
Research	22 (81.48)	12 (100.00)	10 (90.91)	14 (87.50)	14 (73.68)	13 (81.25)	8 (80.00)	93 (83.78)
Administration	1 (3.70)	1 (8.33)	0 (0.00)	1 (6.25)	1 (5.26)	5 (31.25)	0 (0.00)	9 (8.11)
Leadership	1 (3.70)	1 (8.33)	0 (0.00)	4 (25.00)	3 (15.79)	6 (37.50)	2 (20.00)	17 (15.32)
Project coordination	6 (22.22)	6 (50.00)	1 (9.09)	12 (75.00)	8 (42.11)	5 (31.25)	3 (30.00)	41 (36.94)
Communications	1 (3.70)	0 (0.00)	0 (0.00)	3 (18.75)	1 (5.26)	1 (6.25)	2 (20.00)	8 (7.21)
External affairs	0 (0.00)	2 (16.67)	0 (0.00)	0 (0.00)	1 (5.26)	0 (0.00)	0 (0.00)	3 (2.70)
Development	0 (0.00)	0 (0.00)	0 (0.00)	3 (18.75)	1 (5.26)	1 (6.25)	1 (10.00)	6 (5.41)
Management	4 (14.81)	1 (8.33)	0 (0.00)	6 (37.50)	3 (15.79)	3 (18.75)	1 (10.00)	18 (16.22)
Teaching	7 (25.93)	12 (100.00)	10 (90.91)	14 (87.50)	4 (21.05)	15 (93.75)	7 (70.00)	69 (62.16)
Finance	0 (0.00)	0 (0.00)	0 (0.00)	0 (0.00)	0 (0.00)	0 (0.00)	0 (0.00)	0 (0.00)
IT	0 (0.00)	0 (0.00)	0 (0.00)	1 (6.25)	0 (0.00)	0 (0.00)	0 (0.00)	1 (0.90)
Regulatory services	2 (7.41)	1 (8.33)	0 (0.00)	1 (6.25)	0 (0.00)	0 (0.00)	1 (10.00)	5 (4.50)
**KT experience**								
Yes	14 (51.85)	5 (41.67)	7 (63.64)	14 (87.50)	12 (63.16)	11 (68.75)	3 (30.00)	66 (59.46)
No	9 (33.33)	1 (8.33)	1 (9.09)	1 (6.25)	2 (10.53)	4 (25.00)	3 (30.00)	21 (18.92)
Unsure	4 (14.81)	6 (50.00)	3 (27.27)	1 (6.25)	4 (21.05)	1 (6.25)	3 (30.00)	22 (19.82)
**KT activities**								
Written a policy brief	10 (37.04)	3 (25.000)	2 (18.18)	8 (50.00)	11 (57.89)	7 (43.75)	3 (30.00)	44 (39.64)
Written an evidence summary	5 (18.52)	4 (33.33)	4 (36.36)	7 (43.75)	7 (36.84)	5 (31.25)	3 (30.00)	35 (31.53)
Conducted a stakeholder meeting	12 (44.44)	7 (58.33)	5 (45.45)	12 (75.00)	16 (84.21)	10 (62.50)	7 (70.00)	69 (62.16)
Conducted a policy dialogue	3 (11.11)	1 (8.33)	0 (0.00)	6 (37.50)	5 (26.32)	2 (12.50)	2 (20.00)	19 (17.12)
Engaged with an advocacy campaign	5 (18.52)	3 (25.00)	1 (0.09)	7 (43.75)	6 (31.58)	7 (43.75)	2 (20.00)	31 (27.93)
Engaged with policy makers to set priorities	3 (11.11)	4 (33.33)	3 (27.27)	6 (37.50)	9 (47.37)	5 (31.25)	4 (40.00)	34 (30.63)
Developed a video for a policy maker	5 (18.52)	1 (8.33)	0 (0.00)	4 (25.00)	4 (21.05)	2 (12.50)	1 (10.00)	17 (15.32)
Engaged with the media	5 (18.52)	3 (25.00)	2 (18.18)	8 (50.00)	5 (26.32)	8 (50.00)	5 (50.00)	36 (32.43)
Used a KT platform	4 (14.81)	4 (33.33)	2 (18.18)	7 (43.75)	4 (21.05)	0 (0.00)	3 (30.00)	24 (21.62)
Authored or co-authored	8 (29.63)	9 (75.00)	7 (63.64)	11 (68.75)	7 (36.84)	16 (100.00)	9 (90.00)	67 (60.36)
Conducted a systematic or rapid review	6 (22.22)	1 (8.33)	6 (54.55)	5 (31.25)	2 (10.53)	4 (25.00)	4 (40.00)	28 (25.23)
Taught a course on communication, advocacy, stakeholder engagement, or KT	27 (100.00)	12 (100.00)	11 (100.00)	14 (87.50)	19 (100.00)	16 (100.00)	10 (100.00)	109 (98.20)
Worked with a journalist to disseminate information	2 (7.41)	3 (25.00)	0 (0.00)	6 (37.50)	3 (15.79)	4 (25.00)	2 (20.00)	20 (18.02)
Given a presentation at a scientific conference	8 (29.63)	7 (58.33)	6 (54.55)	14 (87.50)	9 (47.37)	15 (93.75)	8 (80.00)	67 (60.36)

Data were missing for some variables, therefore numbers do not always add to the total.

The most common professional foci included research (*n* = 93, 83%), teaching (*n* = 69, 62%) and project coordination (*n* = 41, 36%). A majority indicated that they had experience conducting KT (*n* = 66, 59.46%) while 21 (18.92%) said they did not have experience and 22 (19.82%) were unsure. Participants were provided with a list of KT activities and asked to identify which, if any, they had conducted in the past 3–5 years. Of those who indicated they were unsure if they had experience with KT, 90% (*n* = 20) selected at least one KT activity. Similarly, among those who indicated they had no experience with KT 80% (*n* = 17) selected at least one KT activity.

Individuals who indicated having experience with KT activities (*n* = 66), compared to those who reported no KT experience (*n* = 21), were significantly more likely to have written a policy brief (*n* = 35, 31.53%, *p* < 0.05), conducted a stakeholder meeting (*n* = 47, 42.34%, p-value 0 <0.05), engaged with policy makers to set priorities (*n* = 27, 24.32%, *p* < 0.05), and to have given a presentation at a scientific conference (*n* = 50, 45.05%, *p* < 0.05). Two KT activities also varied significantly by country, “authored or co-authored an article in a peer-review journal” (range 10.45%−23.88%, *p* < 0.01), and “given a presentation at a scientific conference” (range 8.96%−22.39%, *p* < 0.01).

### 3.1 Barriers and facilitators to conducting KT

A list of barriers and facilitators was generated from the literature on KT and findings from the KIIs (11). Facilitators included dedicated personnel, funding, protected time, training, institutional mission/vision/strategy, relationship with ministry members, institutional rewards, and motivated faculty/staff/leadership. Barriers included lack of funding, training, time, networks with the MOH and other stakeholders, awareness of KT, interest from policy makers, understanding the policy process, experience with KT, financial incentives or rewards, and leadership. The most frequently reported facilitators of KT activities at institutional level included motivated faculty (*n* = 64, 57%), dedicated personnel (*n* = 45, 40%) and training (n = 39, 35%) (Table 2).

**Table 2:** Barriers and facilitators, frequencies by independent variables.

	**Top 3 facilitators**			
**Independent variables**	**Motivated faculty**	**Dedicated personnel**	**Training**	**Others** ^*^
	**(*****N** =* **64)**	**(*****N** =* **45)**	**(*****N** =* **39)**	**(*****N** =* **142)**
	***n*** **(%)**	***n*** **(%)**	***n*** **(%)**	***n*** **(%)**
**Country**				
Bangladesh	13 (20.31)	13 (28.89)	8 (20.51)	33 (23.24)
DRC	3 (4.69)	6 (13.33)	6 (15.38)	18 (12.68)
Ethiopia	8 (12.50)	1 (2.22)	6 (15.38)	14 (9.86)
India	6 (9.38)	7 (15.56)	5 (12.82)	24 (16.90)
Indonesia	15 (23.44)	9 (20.00)	3 (7.69)	17 (11.97)
Nigeria	14 (21.88)	6 (13.33)	10 (25.64)	18 (12.68)
Others	5 (7.81)	3 (6.67)	1 (2.56)	10 (7.04)
**Age**				
18–29 years	12 (18.75)	8 (17.78)	4 (10.26)	21 (14.79)
30–49 years	34 (53.12)	23 (51.11)	24 (61.54)	21 (14.79)
50–69 years	15 (23.44)	8 (17.78)	10 (25.64)	27 (19.01)
≥ 70 years	0 (0.00)	1 (2.22)	1 (2.56)	1 (0.70)
**Gender**				
Male	36 (56.25)	26 (57.78)	24 (61.54)	75 (52.82)
Female	27 (42.19)	18 (40.00)	14 (35.90)	65 (45.77)
**KT experience**				
Yes	41 (64.06)	30 (66.67)	23 (58.97)	87 (61.27)
No	12 (18.75)	7 (15.56)	8 (20.51)	25 (17.61)
Unsure	10 (15.62)	8 (17.78)	8 (20.51)	28 (19.72)
	**Top 3 barriers**			
	**Funding**	**Training**	**Time**	**Others** ^**^
	**(*****N** =* **67)**	**(*****N** =* **41)**	**(*****N** =* **41)**	**(*****N** =* **171)**
	***n*** **(%)**	***n*** **(%)**	***n*** **(%)**	***n*** **(%)**
**Country**				
Bangladesh	9 (13.43)	10 (24.39)	12 (29.27)	47 (27.49)
DRC	8 (11.94)	3 (7.32)	2 (4.88)	21 (12.28)
Ethiopia	8 (11.94)	5 (12.20)	1 (2.44)	21 (12.28)
India	11 (16.42)	4 (9.76)	6 (14.63)	28 (16.37)
Indonesia	11 (16.42)	7 (17.07)	8 (19.51)	26 (15.20)
Nigeria	13 (19.40)	7 (17.07)	7 (17.07)	24 (14.04)
Others	7 (10.45)	5 (12.20)	5 (12.20)	9 (5.26)
**Age**				
18–29 years	9 (13.43)	8 (19.51)	8 (19.51)	23 (13.45)
30–49 years	40 (59.70)	19 (46.34)	26 (63.41)	99 (57.89)
50–69 years	15 (22.39)	11 (26.83)	5 (12.20)	40 (23.29)
≥ 70 years	0 (0.00)	1 (2.44)	0 (0.00)	2 (1.17)
**Gender**				
Male	42 (62.69)	20 (48.78)	20 (48.78)	93 (54.39)
Female	24 (35.82)	18 (43.90)	19 (46.34)	68 (39.77)
**KT experience**				
Yes	41 (61.19)	21 (51.22)	26 (63.41)	108 (63.16)
No	12 (17.91)	12 (29.27)	8 (19.51)	28 (16.37)
Unsure	13 (19.40)	7 (17.07)	7 (17.07)	34 (19.88)

^*^Other facilitators included: funding, protected time, institutional mission/vision/strategy, relationship with ministry members, and institutional rewards. ^**^Other barriers included: networks with the MOH and other stakeholders, awareness of KT, interest from policy makers, understanding the policy process, experience with KT, financial incentives or rewards, and leadership.

When stratified by independent variables (country, age, gender and KT experience), training and motivated faculty as facilitators were significantly different by country (*p* < 0.05), selected most frequently by respondents from Nigeria [(training, *n* = 10, 25%), and motivated faculty, *n* = 14, 21%)], and Indonesia (motivated faculty, *n* = 15, 23%). The other facilitators including funding, protected time, institutional mission/vision/strategy, relationships with ministry members, and institutional incentives and rewards, were not significantly different compared by country, age, gender and KT experience.

The most frequently reported barriers included funding (*n* = 67, 60%), training (*n* = 41, 37%), and time (*n* = 41, 37%). Men were significantly more likely to select funding (*n* = 42, 62.69%, *p* < 0.05) and financial incentives or rewards to conduct KT (*n* = 18, 75.0%, *p* < 0.05) as a barrier, while women were significantly more likely to select understanding of the policy process as a barrier (*n* = 13, 48.15%, *p* < 0.05). The other barriers including training, time, network with the MOH and other stakeholders, awareness of KT, interest from policy makers, experience with KT and leadership were not significantly different compared by country, age, gender, and KT experience.

### 3.2 Determinants of readiness factors

The range of standardized raw scores and median for each of the five constructs assessing KT readiness were: Institutional Climate (−1.82–2.23, median = −0.19); Organization Change Efficacy (−2.95–2.42, median = −0.07); Prioritization and Cosmopolitanism (−2.17–2.87, median = −0.11); Self-Efficacy (−2.14–2.54, median = −0.22); and Financial Resources (−1.93–2.47, median = −0.11). Scores from the minimum value to just below the median were assigned a “0” while scores from the median and above were assigned a “1.” We report odds ratios (OR) which indicate the odds of participants reporting above or below the sample median for each construct based on a unit change in the explanatory variable. An OR above 1 indicates increased odds of reporting the factor positively for KT, that is, on average a given individual, with the characteristic captured by the explanatory variable, views the construct as a significant contributor of readiness to conduct KT.

In the bivariate analysis, gender (OR = 0.40; CI: 0.16–0.99; *p* < 0.05) was significantly associated with Organization change efficacy; women compared to men have 60% less likely odds, on average to report this factor as essential for KT. KT experience (OR = 4.09; CI: 1.16–14.40; *p* < 0.05) and professional focus (*teaching*; OR = 0.30; CI: 0.11–0.77; *p* < 0.05) were significantly associated with prioritization and cosmopolitanism. Those with KT experience had 4.09 times increased odds to report this factor as essential for KT activities than those who reported no experience, and those with a professional focus in teaching had 30% less likely odds to report it as essential than those with other foci (including those focused on research). None of the explanatory variables was significantly associated with Institutional Climate, Self-Efficacy and Financial Resources.

In multivariate analysis, country (OR = 4.28; CI: 1.03–16.15; *p* < 0.05) and age (OR = 0.35; CI: 0.03–0.93; *p* < 0.05) were significantly associated with Institutional Climate. Participants from Nigeria were 4.28 times more likely to report Institutional Climate as essential for KT than participants from all other countries. In addition, participants aged 50–69 were 65% less likely to report Institutional Climate as essential for KT than those aged 18–29. Age was also significantly associated with Self-Efficacy. When compared to the youngest age group (18–29), participants aged 30–49 [(OR = 2.98; CI: 1.43–11.53; *p* < 0.05) and 50–69 (OR = 6.25; CI: 1.18–22.78; *p* < 0.05)] were 2.98 and 6.25 times more likely to report Self-Efficacy as essential for KT, respectively. Gender (OR = 0.22; CI: 0.05–0.75; *p* < 0.05) and professional focus (*leadership;* OR = 0.08; CI: 0.00–0.20; *p* < 0.05) were significantly associated with Organizational Change Efficacy. Females were 78% less likely than males, and individuals with a professional focus in leadership were 92% less likely than those reporting other professional foci to report Organizational Change Efficacy as essential to KT activities. Country (*Indonesia*; OR = 3.58; CI: 1.35–20.31) was the only variable significantly associated with Prioritization and Cosmopolitanism; participants from Indonesia were 3.58 times more likely to report this factor as essential for KT than participants from other countries. A professional focus in project coordination vs. all other professional foci (OR = 0.36; CI: 0.10–1.33; *p* < 0.05) was significantly associated with Financial Resources. Those with a focus on project coordination were 64% less likely to report Financial Resources as essential for KT than participants with other professional foci (research, administration, leadership, communication, external affairs, development, management, teaching, finance, IT, and regulatory services). The odds ratios for the multivariable analysis are shown in Table 3. The models explained between 10 and 31% of the variance in explaining each factor.

**Table 3:** Final factor regression model.

**Variable**	**Factor 1:**	**Factor 2:**	**Factor 3:**	**Factor 4:**	**Factor 5:**
	**Institutional climate**	**Organization change efficacy**	**Prioritization & cosmopolitanism**	**Self-efficacy**	**Financial resources**
	**Odds Ratio of scoring above the median readiness score with respect to each Factor (95% CI)**				
**Age**					
18–29 years (Ref.)					
30–49 years	0.85 (0.13–5.36)	2.25 (0.21–23.83)	0.43 (0.06–3.15)	2.98 (1.43–11.53)^*^	1.48 (0.25–8.80)
50–69 years	0.35 (0.03–0.93)^*^	2.46 (0.14–42.59)	0.86 (0.07–11.26)	6.25 (1.18–22.78)^*^	1.26 (0.14–11.69)
**Gender**					
Male (Ref)					
Female	1.05 (0.34–3.26)	0.22 (0.05–0.75)^*^	0.67 (0.22–2.07)	0.10 (0.34–2.94)	1.65 (0.58–4.71)
**Country**					
Bangladesh	0.23 (0.02–2.96)	6.62 (0.32–138.36)	0.39 (0.03–5.02)	3.19 (0.22–46.05)	0.85 (0.11–6.77)
DRC	0.59 (0.04–8.05)	0.16 (0.01–2.65)	0.34 (0.03–5.02)	0.80 (0.06–10.53)	1.71 (0.21–14.07)
Ethiopia	1.13 (0.08–15.56)	0.19 (0.01–2.94)	1.45 (0.10–21.70)	0.87 (0.06–11.66)	0.42 (0.05–3.57)
India	1.22 (0.09–17.61)	0.06 (0.00–1.10)	0.14 (0.01–2.35)	2.82 (0.19–42.39)	0.84 (0.12–5.75)
Indonesia	0.16 (0.01–2.03)	0.53 (0.04–7.57)	3.58 (1.35–20.31)^*^	0.5 (0.04–5.87)	0.54 (0.07–3.89)
Nigeria	4.28 (1.03–16.15)^*^	0.35 (0.03–4.88)	0.31 (0.02–4.06)	0.74 (0.07–8.28)	1
Others (Ref)	1	1	1	1	1
**KT experience**					
No (Ref.) Yes	2.58 (0.54–12.25)	16.51 (1.85–147.71)	4.33 (0.90–20.74)	0.72 (0.17–3.13)	0.42 (0.09–1.84)
Unsure	2.39 (0.38–15.20)	6.34 (0.80–50.42)	2.95 (0.48–18.33)	0.75 (0.13–4.42)	0.93 (0.14–5.96)
**Professional focus**					
Research	–	0.35 (0.04–2.94)	–	0.27 (0.05–1.56)	–
Administration	0.62 (0.04–10.57)	3.57 (0.20–64.00)	3.21 (0.32–32.01)	0.45 (0.04–5.32)	–
Leadership	0.18 (0.02–1.82)	0.08 (0.00–0.20)^*^	0.19 (0.02–1.52)	4.87 (0.57–41.27)	1.29 (0.29–5.77)
Project coordination	0.38 (0.08–1.75)	–	–	–	0.36 (0.10–1.33)^*^
Communications	–	14.68 (0.32–671.75)	27.60 (0.26–294.12)	–	–
External affairs	–	2.04 (0.48–87.49)	–	1.95 (0.06–61.26)	–
Development	4.60 (0.28–75.27)	–	0.24 (0.01–5.85)	3.73 (0.24–57.81)	–
Management	0.42 (0.05–3.80)	9.66 (0.63–148.44)	3.63 (0.52–25.13)	0.43 (0.06–3.20)	–
Teaching	–	2.76 (0.34–22.31)	–	0.43 (0.08–2.38)	0.92 (0.21–4.09)
Finance	–	–	–	–	–
IT	–	–	–	–	–
Regulatory services	–	–	1.19 (0.08–18.84)	2.44 (0.15–38.98)	3.32 (0.19–56.92)
All other foci (Ref)					

R^2^ for Factor 1= 20%; R^2^ for Factor 2 = 31%; R^2^ for Factor 3 = 20%; R^2^ for Factor 4 = 14%; R^2^ for Factor 5 = 10%. “-” indicates this variable was not included in the model for a given Factor. ^*^Significant at ≤ 0.05.

### 3.3 KT index

The range of scores for the KT index was −2.98–3.08 and the median cut off was 2.33. Scores from the minimum value to just below the median were assigned a “0” while scores from the median and above were assigned a “1.” We report ORs for each independent variable in the model. An OR above 1 indicates participants who scored above the median, associated with an increase in odds for reporting readiness to conduct KT.

We performed bivariate and multivariate logistic regression on the KT index. In the bivariate analysis, KT experience (OR = 2.86; CI: 1.04–7.83; *p* < 0.05), professional focus (*leadership*; OR = 0.28; CI: 0.10–0.83; *p* < 0.5), barriers (*training;* OR = 0.44; CI: 0.20–0.97; *p* < 0.05), and facilitators (*training;* OR = 0.45; CI: 0.20–0.99; *p* < 0.05 and *motivated faculty;* OR = 0.31; CI: 0.13–0.70; *p* < 0.05) were significantly associated with readiness to conduct KT. Those reporting KT experience were 2.86 times more likely to report readiness to conduct KT compared to those who reported no experience with KT. Participants in leadership were 72% less likely than those with other professional foci to report readiness. Participants who reported training as a top barrier compared to all other barriers were 56% less likely to report readiness. Participants who reported training and motivated faculty as facilitators compared to all other facilitators were 55% and 69% less likely to report readiness for KT, respectively.

In multivariate analysis, KT experience (OR = 9.07; CI: 1.60–51.58; *p* < 0.05) and two facilitators (*dedicated personnel;* OR = 0.23; CI: 0.07–0.80; *p* < 0.05 and *motivated faculty*; OR = 0.23; CI: 0.06–0.92; *p* < 0.05) were significantly associated with the index. Participants who indicated experience with KT were 9.07 times more likely to report readiness for KT than those with no experience. Participants who considered dedicated personnel and motivated faculty to be top facilitators vs. other facilitators were 77% less likely to report readiness for KT.

All odds ratios and confidence intervals for the KT index multivariable model are shown in Table 4.

**Table 4:** KT index regression model.

**KT index**	**Odds Ratio for scoring above the median readiness score (95% CI)**
**Age group**	
18–29 years (Ref.)	
30–49 years	1.50 (0.23–9.85)
50–69 years	1.32 (0.11–15.63)
**Gender**	
Male (Ref) Female	0.61 (0.19–1.97)
**Country**	
Bangladesh	3.66 (0.29–47.03)
DRC	3.82 (0.20–72.46)
Ethiopia	2.08 (0.11–40.18)
India	1.79 (0.14–22.29)
Indonesia	1.11 (0.08–15.36)
Nigeria	1.95 (0.13–29.92)
Others	1
**KT experience**	
No (Ref) Yes	9.07 (1.60–51.58)^*^
Unsure	2.24 (0.42–12.04)
**Professional focus**	
Research	1.05 (0.21–5.20)
Administration	4.31 (0.32–57.21)
Leadership	0.14 (0.15–1.28)
Project coordination	0.67 (0.19–2.42)
Communications	1.57 (0.10–23.80)
Management	1.13 (0.16–8.20)
Teaching	0.36 (0.05–2.47)
Regulatory services	2.07 (0.09–47.21)
All other foci (Ref)	
**Facilitators**	
Dedicated personnel	0.23 (0.07–0.80)^*^
Funding	0.67 (0.21–2.20)
Protected time	3.08 (0.48–19.82)
Institutional rewards	1.39 (0.18–10.50)
Motivated faculty, staff, & leadership	0.23 (0.06–0.92)^*^
All other facilitators (Ref)	
**Barriers**	
Time	1.72 (0.50–5.91)
Understanding the policy process	0.95 (0.22–4.05)
Experience with KT	2.06 (0.46–9.18)
Financial incentives or rewards	0.99 (0.25–3.93)
Leadership	0.87 (0.19–3.99)
All other barriers (Ref)	

R^2^ = 26%. ^*^Significant at ≤ 0.05.

## 4 Discussion

Most survey participants, even those who reported not having experience with KT, indicated that they conduct KT activities. Clearly many members of academic institutions conduct KT but fewer conduct it systematically and with intention.

This study identified four determinants (age, gender, professional focus, and country) that are uniquely associated with five different underlying factors of institutional readiness to conduct KT.

For KT readiness, institutional climate (organization level) was on average more relevant to younger individuals while self-efficacy (individual level) was more relevant for those in mid- and older age groups. Institutional climate, a concept first described by Klein and Sorra (32), represents a shared receptivity for change and the extent to which that change will be supported and rewarded by the institution. Younger individuals, more junior in their profession, likely rely more on internal support to conduct KT, particularly if they have not yet established their own external networks and resources. Self-efficacy, conversely, is an individual-level factor, referred to as an individual's beliefs in their capability to executive behaviors necessary to produce specific achievements (33). This concept represents a perceived internal control over one's behavior and environment. Studies have demonstrated the interconnection between improved self-efficacy and organization context. Gist and Mitchell described key determinants to self-efficacy including personal mastery and vicarious experience, both of which are attained over time and through experience (34). It is reasonable to expect that older individuals, with greater levels of mastery, would feel more control over their environment and have increased confidence in their abilities to conduct KT. Organization change efforts should ensure dialogue between these groups of professionals, highlighting areas for institutional improvement (of high relevance to younger groups which may be controlled by older groups). This can represent opportunities for KT-specific mentorship which has been shown to provide credible, tailored information, on on-going and as-needed bases (35).

On average, organization change efficacy (organization level) was a more significant contributor to KT readiness for men than women and less significant for those in leadership than those with other professional foci. While supportive leadership is not the only ingredient needed to promote organizational change, it is well-documented as an important one (36). Consistent messaging and actions from institutional leaders can promote a shared vision for change among organizational members (37). Leaders who effectively promote organizational change are also frequently described as having increased self-efficacy, bringing to bear their perceptions of environmental control and personal motivation to enhance their organizations' mission and influence other organizational members (38). It is imperative to continuously engage members of leadership to influence organizational level change and create enabling environments for junior KT researchers. Again, KT mentorship programs may provide an avenue for ongoing engagement and could improve the KT environment (35).

Institutions should similarly note the influential role of women leaders in affecting organizational change and improving organizational change efficacy (39). Women continue to be less represented in leadership in academic science, technology, engineering, mathematics, and medicine (40). Encouraging women in leadership roles is especially relevant for KT in global health since women experience a disproportionate burden of disease and women represent most of the health workforce (but only 25% of leadership) (41). Current literature on the role of gender in knowledge translation and knowledge management specifically is limited but suggests that gender is an important influencer (42). Women globally face participation challenges, have less access to technology, and experience gender biases including in publication (43), a critical approach to knowledge sharing for academic institutions. This lack of representative leadership may contribute to women participants being less likely to feel a shared sense of confidence in collectively implementing change for KT. Capacity building efforts that seek to improve organizational change efficacy for KT should consider the intersection of gender and leadership as predictors for this factor and design interventions that support and encourage women in leadership roles.

Financial resources were a less significant contributor of readiness for KT to those in coordination roles than others. Coordination can encompass many tasks though may be viewed as a more ‘junior' focus. It may be that these respondents are less aware of or engaged in budgeting and procuring and managing financial resources. Our research identified funding as one of the top three barriers to conducting KT which is consistently supported by the literature, particularly for LMICs (11, 44–46). Those in program coordination should be introduced to broader aspects of KT activities including the role of funders, and available internal and external resources for KT. The importance of financial resources should also be considered for designing and budgeting KT activities from the beginning of any project, as well as for systematic evaluation of KT strategies.

Country context significantly determined some factor scores but not others. Participants from Nigeria were more likely to report institutional climate as a contributor to KT than individuals from other countries while Indonesian participants were more likely to report prioritization and cosmopolitanism as a significant contributor than individuals from other countries. Data from the KIIs suggest these findings may be related to where each institution is on the spectrum of development. One participant from Indonesia described this process over time, “*So in previous times, it was more building capacity for research and good quality research. And then we moved to the stage where people are pushing more for at least disseminating in terms of scientific publication, scientific journals. We are still at that stage, but now more and more people are also questioning what are the impacts of research that we are doing. So, more and more people are pushing for knowledge translation.”* As KT increasingly becomes a priority for internal and external stakeholders, strong networks and aligned institutional priorities may have a larger impact on readiness than institutional climate (which may have already shifted positively to prioritize KT). Capacity building approaches should account for organizational evolution, designing strategies that facilitate growth among the factor most relevant for the given stage of change.

Interestingly, KT experience was not significantly associated with any of the five constructs in the multivariate analysis. However, when all scores for these constructs were combined to create an index, those with KT experience were 9.07 times more likely to report readiness for KT scores than those who said they had no experience. This demonstrates the value that experience can have on overall readiness to conduct KT (regardless of underlying factor) and highlights the need to continuously engage those with experience to serve as mentors and develop the skills of less experienced team members. It should be noted that there may have been confusion among participants about what KT is and whether they have done KT. Data shows a discrepancy between those who indicated they had no experience in KT or were unsure, and the selection of KT activities they had conducted in the past 3–5 years. Participants were first asked to define KT in their own words and then presented a list of KT-related activities. When the activities are described, most individuals realized they had done or are currently doing KT in some form, though they might not have called it that initially. Many KT activities are organically conducted by most academic researchers. It is important however to create systematic KT processes with an end goal in mind, much like with research objectives and aims. These processes can be incorporated at the organizational level (e.g., strategic plans) and individual level (e.g., career development plans), providing a more nuanced road map for researchers with demonstrated buy-in by the institution.

Each of these findings can be used to inform and adapt strategies designed to improve readiness to conduct KT. For example, twining faculty with more KT experience with faculty with less experience is a potential strategy that can support readiness to do KT in academic institutions in LMICs (47). Other strategies can be developed around dedicated personnel units to conduct KT as suggested by this study. Proctor et al. argue for a systematic approach to developing and reporting implementation strategies to avoid inconsistencies and promote replication (48). They offer 7 domains for specification: actor(s), action(s), target(s), temporality, dose, outcome(s) affected, and justification. The determinants of each KT factor and KT index can serve as an initial guide to completing the domains in this framework through the selection of relevant targets (e.g., men or women or those with different professional foci). Specifying each strategy across these domains also provides guidelines for developing well-defined metrics that can be used for robust evaluations, thus informing the effectiveness of such strategies in improving readiness for KT.

### 4.1 Strengths and limitations

The development of the assessment tool discussed in this research was rooted in organizational change theory, using domains, constructs, and items from validated tools. We conducted the research across multiple contexts in both Africa and Asia to capture factors and their determinants generalizable to academic institutions based in different LMIC settings. A significant limitation of this research is that the data used to derive the determinants is the same data set used to establish the five underlying factors of readiness. However, these five factors are not necessarily de-novo. Many are well-established in organizational change theory and are now adaptable to LMIC contexts. Another limitation is the possible presence of selection bias if participants whose responses were dropped were similar in some way (e.g., all of a similar age group, from the same country, or with similar professional foci). We also recognize that the sample size for this research is small and did not allow for exploring interactions in the regression models. This is an important area for future research.

## 5 Conclusions

Age, gender, country, professional focus, and experience with KT are all determinants of different KT factors and overall KT readiness, and different factors of KT readiness are relevant for members of different professional and age groups. These findings may suggest that mentorship models that enable open dialogue and sharing between groups could increase KT readiness and engagement of organizational leaders as champions for change could facilitate KT, and institutions should consider policies that foster women leaders in KT.

## Data availability statement

The raw data supporting the conclusions of this article will be made available by the authors, without undue reservation.

## Ethics statement

Ethical approval was not required for the study involving humans in accordance with the local legislation and institutional requirements. The studies were conducted in accordance with the local legislation and institutional requirements. Written informed consent to participate in this study was not required from the participants in accordance with the national legislation and the institutional requirements.

## Author contributions

AK: Conceptualization, Investigation, Writing—original draft. AR: Formal analysis, Investigation, Methodology, Writing—review & editing. OA: Conceptualization, Supervision, Writing—review & editing.
